# The Missing Link between Juvenile Delinquency and Pediatric Posttraumatic Stress Disorder: An Attachment Theory Lens

**DOI:** 10.5402/2012/134541

**Published:** 2012-06-14

**Authors:** Pooja L. Amatya, Drew H. Barzman

**Affiliations:** Division of Child and Adolescent Psychiatry, Cincinnati Children's Hospital, Cincinnati, OH 45229-3026, USA

## Abstract

The present paper reviews pediatric posttraumatic stress disorder, emphasizing the relational basis of the disorder and highlighting the missing link between juvenile delinquency and trauma. The first part of the paper defines trauma and the diagnostic criteria for PTSD, noting child-specific features. The second part reviews the literature emphasizing the relational and attachment relevant nature of trauma. The third part explores psychological mechanisms for how attachment relations could affect trauma responses. Attachment relations (1) shape core schemas of the world, others, and the self and (2) foster emotional engagement or disengagement, both of which have been associated with traumatic responses. The most empirically supported pediatric trauma treatment, trauma-focused cognitive behavioral therapy (TF-CBT), acknowledges the attachment figure's influence and includes treating and training the parent and conjoint child-parent discussion. The next section reviews the noteworthy link between juvenile delinquency and trauma history. More awareness of trauma and PTSD in children and adolescents is recommended to effectively address juvenile delinquency. The review ends with a few helpful points for practicing pediatricians regarding childhood trauma.

## 1. Introduction

Trauma and PTSD in children occur in the context of the ongoing parent-child relation, and this lens may offer insights to the correlations which previous studies have uncovered, integrating the findings and adding a fuller contour to the picture of pediatric PTSD. This paper aims to review and integrate an understanding of PTSD in children and adolescents in an attachment context and organizes the article in several sections: defining PTSD in children; a review of parental influences on pediatric PTSD; psychological mechanisms for how attachment relations affect trauma responses; the link between PTSD and juvenile delinquency; a few take-away points for pediatricians.

## 2. Defining PTSD in Children

Trauma is a profound experience of the loss of security and welfare evoking feelings of fear, helplessness, horror, and possibly disorganized agitation in children [1]. The description of PTSD in the Diagnostic and Statistical Manual of Mental Disorders has increasingly taken into account child-specific features of PTSD, and PTSD has evolved from a disorder first associated with Vietnam veterans in the 1970s [2] to one which has now been well documented in the general population at an estimated 7.8% [3] and in specific groups of children, particularly high-risk children such as adolescent delinquents, though an estimate of PTSD in the population of children and adolescents is still forthcoming. In fact, Ruchkin et al. [4] found 25% of adolescent delinquents met DSM-III PTSD criteria and 42% fulfilled partial PTSD criteria, percentages comparable to and exceeding those found in the population of Vietnam veterans [5], suggesting the vulnerability of childhood or adolescent experiences and the subjective nature of trauma. 

The latest DSM-IV-TR outlines the diagnostic criteria for PTSD following the occurrence of a traumatic event, including symptoms for longer than one month of (1) reliving the trauma, (2) avoiding associations related to the trauma, and (3) increased arousal resulting in “clinically significant distress or impairment in social, occupational, or other important areas of functioning” [1]. The description is keen to note the impact of childhood development on the triad of symptoms; for instance, in children, the response to the trauma may be expressed as disorganized and agitated behavior rather than intense fear, horror, and helplessness, and the reexperiencing may be expressed through repetitive play with traumatic themes, frightening dreams without recognizable meaning, and trauma-specific reenactment [1].

Children may suffer from trauma in many significant ways including developmental delays; numerous social, behavioral, and academic difficulties; somatic complaints; lower self-esteem; attachment issues [6]. Thus, PTSD should be understood as one clinically significant effect of trauma that may occur in the context of and be linked to many other subtle or explicit effects on the child's functioning and development. 

## 3. Review of Parental Influences on Pediatric PTSD

To more deeply understand the nature of post traumatic stress disorder (PTSD), including the impact of the etiological event of the trauma and the manifestation and trajectory of its symptoms, some scholars have turned to the influence of attachment figures and attachment styles on PTSD. This focus occurs in the larger context of the growing interest in emotions and their social function in the psychological sciences [7] and psychopathology [8]. Unhealthy attachment styles have been correlated to greater PTSD symptoms in adults [9, 10], and Scheeringa and Zeanah [11] review 17 studies which consistently found undesirable parental variables linked to elevated child PTSD or other detrimental symptoms following trauma. 

Other pools of research which suggest the influence of parents on pediatric PTSD include papers on the interpersonal aspects of trauma and PTSD [12], the effects of abuse and neglect on development [6], social support and PTSD [13], parent psychopathology and child behavioral problems [14], parent child reporting of child PTSD symptoms [15], and the benefits of parent participation in child psychotherapy [16]. 

Much of the highlighted literature is guided by the primacy of the parent-child relationship and the strong influence of parent variables and behaviors on child outcomes. Research in developmental [17, 18] and evolutionary psychology [19] as well as neuroanatomy and neuroendocrinology [20] has converged in validating the safety and well-being that parenting can provide, as well as the negative consequences caused by attachment disruptions, an intuitive result of the primacy of that relation. And parenting literature, which pertains more to parenting in a normative context, has consistently demonstrated correlations between certain types of parenting and child competence; for instance, authoritative parenting characterized by high emotional responsiveness as well as high behavioral goals, yet lower psychological control [21], has been associated with high child competence [22, 23].

## 4. Psychological Mechanisms for How Attachment Relations Affect Trauma Responses

What psychological mechanisms may explain how attachment relations affect traumatic responses? It is interesting that both literature in trauma and that in attachment relations discuss the role of (1) core schemas and (2) emotional engagement as relevant psychological mechanisms. 

First, if attachment relations shape core schemas, and PTSD is caused by negative schemas of the self and world formed through trauma, then it is plausible that attachment figures can play a role in influencing PTSD. 

Schemas are a conceptual framework based on life experiences that help the individual organize information and interpret and adapt to the environment. Trauma can cause damage either by negatively changing one's positive schemas of one's self as competent and the world as safe or by priming existing knowledge of one's self as incompetent and the world as dangerous [24]. Though the first pathway is intuitive, the latter pathway applies to trauma victims with previous traumatic experiences and pretrauma psychological disturbances. Foa [24] cites studies which provide evidence for both these pathways, such as an earlier study by the author which found that individuals who had PTSD 3 months after trauma showed a less positive view about the world and themselves just after the trauma than those who recovered ([25] as cited in [24]). Another paper described how earlier victimization and preexisting biopsychosocial problems delay rape recovery [26].

Attachment theory founder Bowlby [17] conceived of attachment as an evolutionarily crucial form of behavior distinct from feeding and sex with the purpose of gaining proximity, care, and protection from the attachment figure, the infant caregiver or mother. Bowlby [27] believed that repeated interactions with attachment figures shaped children's dynamic and increasingly complex internal working models of the world, self, and important relations; comforting behavior by attachment figures results in internal working models of the self as worthy and the world as safe versus the self as unworthy and the world as dangerous. 

According to developmental psychologist Jean Piaget, who is commonly known for introducing and popularizing the term schema, childhood was a time of rapid schema development. Thus, behaviors by attachment figures (as well as childhood trauma) should have a stronger impact during this time in schema formation, providing the foundational schema which can be further tweaked. 

Following the line of thought of the core schema theory, attachment figures that strengthen the child's schemas of the self as competent and the world as safe can play a role in mitigating the negative effects of trauma and the likelihood of PTSD. 

Second, if emotional engagement with the trauma facilitates trauma resolution, then the healthy attachment relationship, which can elicit strong feelings of engagement and comfort, should help process trauma feelings and mitigate PTSD. Conversely, trauma occurring in the context of an unhealthy attachment relationship which hinders emotional engagement with the trauma should increase PTSD. 

Research points to early emotional engagement with the trauma as crucial for trauma processing and recovery and emotional withholding, avoidance, or disassociation as linked to PTSD severity [28–30]. Indeed, complex PTSD, occurring after long-term trauma, is more correlated with dissociative symptoms and also more difficult to treat [31]. 

Attachment relations can play a critical role in emotional engagement with the trauma. First, it is within the context of the attachment relation that infants first learn about emotional engagement, as both the receiver and giver of it, developing styles of engagement (secure, anxious avoidant, or anxious ambivalent) based on patterns of the attachment figure's sensitivity, responsiveness, timeliness, and availability [32]. For the infant, physical bonding is an essential aspect of emotional engagement; in lieu of verbal vocabulary, touch can communicate emotions and information to the infant based on the qualities of the interaction [33] and facilitate state regulation and coping with stress and arousal [34].

Ainsworth [32] found that the most significant commonality for those infants developing an avoidant style was mothers with an aversion for physical contact. Disengagement is then a strategy by the infant to cope with the mother's expected rejection and hostility. The attachment relation is built by interactions of emotional engagement or disengagement. In fact, an independent emotional system characterized by feelings of calm, comfort, and emotional engagement, with a discrete system of brain circuits, evolved to support attachment in human evolution for parenting, just as separate emotional systems of lust and attraction evolved to foster the other two primary behaviors of mating and reproduction [35]. 

Attachment theory holds that this attachment style, though first shaped by the attachment figure, extends beyond the primary relation and is correlated with later responses and psychopathology [36]. On the same note, Dieperink et al. [37] found that adult former prisoner war veterans with secure attachment styles scored significantly lower on measures of PTSD than did those with insecure styles, and that PTSD symptom intensity was predicted better by attachment style than trauma severity. This resonates with the idea that the style of emotional engagement, first shaped by the attachment figure, is used to process later events such as trauma. 

Second, attachment figures can help children emotionally engage with and process the relevant trauma. Though attachment style is relevant throughout the lifespan, the facilitation of emotional engagement by attachment figures like parents is especially pertinent for children facing childhood trauma; attachment theory holds that attachment behaviors are most visible during early childhood and during times of distress and uncertainty [17], both of which are relevant for pediatric trauma. The evolutionary purpose of attachment is protection, and it is in such moments requiring that the attachment dynamic becomes more salient [17], offering a reassurance distinct from and beyond social support or peer approval [32]. Attachment figures can help children emotionally engage with their presence, providing a secure base [32] and reliable boundaries amidst unpredictable and disruptive situations, through attentiveness to and inquiries of the child's experiences and needs; nonjudgment and acceptance of strong feelings; offering empathetic validations. Attachment figures can also help with sense making of traumatic events, negotiating implications of traumatic situations to less distressing ones.

The most empirically supported pediatric trauma treatment, trauma-focused cognitive behavioral therapy (TF-CBT), incorporates all of the previously discussed aspects, aiming to support positive core schemas and correct maladaptive ones, foster emotional engagement, and strengthen the parent child bond [38]. This treatment for children of ages 3 to 17 suffering from trauma-related negative feelings and behaviors consists of implementing a therapeutic sequence summarized by the acronym PRACTICE, including psychoeducation about trauma; relaxation skills; affect or emotional processing and management; cognitive processing and management; trauma narration and processing; conjoint child-parent discussion; finally, enhancing well-being, safety, and development [38]. It is important to note that the parent undergoes treatment and training along with the child (in individual sessions for the first four phases, preparing the parent to be as supportive as possible following the trauma narration and later joint parent-child sessions). 

The complexity of trauma situations and degree and nature of parent involvement in the trauma, from less involved, witness, victim similar to the child, enabler, or perpetrator, will affect the parent-child dynamic, parent and child symptomatology, and healing of the trauma. As discussed in the literature review, relational traumas are more influential, and thus, traumas in which parents are involved in a more negative light will likely be more challenging. However, as discussed above, TF-CBT provides a general method for addressing childhood trauma, and with the assumption that the involved caregiver is not a current threat to the child, it utilizes the strength of the parent-child attachment dynamic in facilitating cognitive and emotional trauma processing for healing. 

While TF-CBT is empirically supported [38], it is less certain which phase or combination of phases lends to its efficacy. For instance, Deblinger et al. [39] tested TF-CBT variations (8 weeks versus 16 weeks and trauma narration vs. no trauma narration) and found that though all variations effectively improved symptoms, parenting skills, and children's personal safety skills, the 8-week trauma narrative version most decreased parent and child abuse-related anxiety, while the 16-week no narrative condition most increased effective parenting practices and decreased child externalizing problems. Perhaps a trade-off occurs in how time is utilized in therapy, with focusing on parenting skills decreasing the child's acting-out behaviors, while talking about the trauma may relieve some of the anxiety associated with it. Overall, the paper seems to suggest that healing occurs more through the processing of emotions and beliefs in context of the strengthened parent-child bond rather than formal trauma narration or an extended length of therapy. 

## 5. Societal Implications of Pediatric PTSD: Juvenile Delinquency

As an anxiety disorder, one may imagine PTSD to be a source of strife for the victim and perhaps close a circle of contacts. However, rather than being a quietly contained disorder, the violence and chaos experienced by the victim through trauma may manifest as outward acts of aggression, delinquency, and conduct disorder; conduct disorder behaviors and the like may be considered as symptoms of PTSD, rather than isolated problems. Ryan and Testa's [40] finding resonates with this point: substantiated victims of maltreatment averaged 47% higher delinquency rates relative to nonabused children. Similarly, Steiner et al. [41] found 31.7% of severely delinquent juvenile offenders with PTSD and 20% meeting partial criteria, compared to the 9.3% in nonclinical sample of youth. 

In youth with conduct disorder, Reebye et al. [42] found PTSD more frequently in girls, who reported more sexual assault trauma than the physical trauma and witnessing death described by boys. Similarly, in the general population at large, the National Comorbidity Survey found that although men were more likely to experience one trauma overall, women were both more likely to experience a trauma more correlated with PTSD in that gender (like rape for females) and more affected by traumas in general, with women more than twice as likely to develop PTSD after a described trauma [3]. This point is important because the number of crimes by female adolescents rose 23% between 1989 and 1993 compared to the 11% in males—with severely violent crimes rising by a staggering 55% in females compared to 33% in males [43], and trauma in girls could help make sense of this increase.

The environmental factors conducive to juvenile delinquency such as parental psychopathology, drug use, and criminal activity; neighborhoods with criminal subculture, low participation, and frequent mobility among residents; poor school conditions with chaotic environments [44] are also likely factors conducive to trauma and characteristic of environments that are less supportive following trauma. Furthermore, once trauma has occurred, PTSD itself devoid of these environmental influences can lead to juvenile delinquency. Steiner et al. [41] theorizes that PTSD creates possible pathways for aggression in four distinct ways: arousal-driven interference with relaxation and learning, inappropriate reliving-driven hostile reactions, intrusion-driven continuous trauma reenactment, and unnecessary avoidance of triggers. 

Thus, children in the previously mentioned negative environments who have also experienced trauma are at greatest risk for juvenile offending, and care should be taken to view aggressive actions in context of trauma history and environment rather than as merely innately malicious acts. Individual risk factors associated with juvenile delinquency such as low social conformity, drug use, low self-esteem, antisocial attitudes, noncompliance, and poor school achievement [44] are all eerily familiar with the summary of research findings on the impact of childhood abuse and neglect [6], suggesting that traumatized children are also at greater risk for delinquency due to the impact of the trauma to one's attitudes and skills. 

Tarolla et al. [44] describe juvenile delinquency as one of our most urgent social issues, with offenders using a large share of child welfare, juvenile justice, special education, and mental health resources. Understanding, preventing, and treating childhood PTSD are of importance not only to emotionally benefit the victim, the primary focus of mental health professionals, but also to avoid costs and reap benefits on a societal level throughout the course of the child's development into adulthood. The less than established effectiveness of many of the available juvenile delinquency prevention and treatment plans [45] may be due to the lack of relational and trauma focus of these plans. Echoing this point, Mulvey et al.'s paper [45] finds longer community-based interventions that improve family functioning, incorporate behavioral intervention, and improve social networks more useful for treatment. 

## 6. A Few Helpful Points for Pediatricians

The child's advocates include the family, teachers, pediatrician, and mental health care specialists such as social workers, therapists, psychologists, and psychiatrists, and in light of the previous research, measures taken by these members to resolve trauma are in themselves a preventative measure against juvenile delinquency. Though mental health care specialists will be responsible for the formal psychological assessment and treatment, pediatricians aware of trauma symptoms and treatment will have a more comprehensive understanding of the patient and can also effectively make referrals if needed. 

Based on this paper, a few take-away points regarding childhood trauma for pediatricians include the chaotic environmental factors conducive to both trauma and juvenile delinquency described in the previous section; the earlier described DSM-IV criteria for PTSD, noting child-specific features; the fact that trauma can affect the child without the child meeting full PTSD criteria and that these effects may subtly or explicitly affect the child's functioning and development, manifesting as developmental delays, behavioral or academic difficulties, or somatic complaints and may also resemble symptoms traditionally associated with ADHD, conduct disorder, or juvenile delinquency (see [6] for a fuller description of the developmental effects of childhood maltreatment); findings that traumas of an interpersonal nature are more associated with PTSD [12]; the potential for the healthy attachment relation to heal trauma. Further, a mental health specialist skilled in TF-CBT, the most empirically supported treatment for childhood trauma, would be the first line of recommended treatment. 

Understanding trauma history and strengthening attachment relations will be a critical piece in addressing and healing delinquent children and adolescents.
